# Hypermethylation-mediated silencing of RASD1 drives multiple myeloma pathogenesis

**DOI:** 10.1007/s44313-026-00125-6

**Published:** 2026-02-02

**Authors:** Chenfeng Yi, Nana Ren, Yuxi Cai, Jiajia Zhang, Yonghuai Feng

**Affiliations:** 1 Department of Hematology, Dongguan People’s Hospital (Southern Medical University, The Tenth Affiliated Hospital), Dongguan, Guangdong 523059 China; 2 Department of Hematology, Affiliated Hospital of Zunyi Medical University, Zunyi, Guizhou 563000 China; 3 Department of Internal Medicine I, Affiliated Hospital of Jinhua University of Vocational Technology, Jinhua, Zhejiang 321000 China

**Keywords:** Multiple myeloma, RASD1, DNA methylation, Epigenetics, Tumor suppressor gene

## Abstract

**Background:**

The role of Ras-related dexamethasone-induced 1 (RASD1) in multiple myeloma (MM) pathogenesis remains unclear. This study investigated the expression profile, clinical significance, and epigenetic regulation of RASD1 in MM.

**Methods:**

Bone marrow samples were collected from 26 newly diagnosed patients with MM and 8 healthy controls. RASD1 messenger RNA (mRNA) and protein expression were analyzed using reverse transcription quantitative polymerase chain reaction (RT-qPCR) and immunohistochemistry, respectively. DNA methylation status was assessed via methylation-specific PCR (MSP). The U266 MM cell line was treated with the demethylating agent decitabine (DAC) to evaluate its effects on RASD1 expression and apoptosis.

**Results:**

RASD1 mRNA and protein expression were significantly downregulated in patients with MM compared to healthy controls (*P* < 0.001). Low RASD1 mRNA levels correlated significantly with advanced DS stage, anemia, hypercalcemia, and elevated M-protein concentrations (*P* < 0.05). The receiver operating characteristic curve indicated that RASD1 mRNA expression was a robust discriminator between patients with MM and healthy individuals (area under the curve = 0.882, sensitivity = 100%, specificity = 75%). MSP analysis revealed RASD1 promoter hypermethylation in patients with MM, whereas controls exhibited hypomethylation. Treatment of U266 cells with DAC restored RASD1 expression and significantly increased apoptosis compared with controls (12.08% vs. 5.04%, *P* < 0.01).

**Conclusion:**

RASD1 is frequently silenced in MM through promoter hypermethylation. This epigenetic inactivation is associated with adverse clinical features and enhanced cell survival, supporting a tumor suppressor role for RASD1 in MM pathogenesis.

## Introduction

Multiple myeloma (MM) is a malignant neoplasm of clonal plasma cells and represents the second most common hematological malignancy [1]. Despite therapeutic advances, including the introduction of novel agents and stem cell transplantation, MM remains largely incurable due to the inevitable development of relapsed or refractory disease [2]. A deeper understanding of its molecular pathogenesis is crucial for identifying new therapeutic targets.

Ras-related dexamethasone-induced 1 (RASD1) is a member of the Ras superfamily of small G-proteins. Unlike its well-characterized oncogenic counterparts (e.g., K-Ras, H-Ras), RASD1 exhibits context-dependent roles in cancer and can function as a tumor suppressor, exerting growth-inhibitory effects in lung adenocarcinoma and breast cancer cells [3, 4]. Conversely, it may promote progression in malignancies such as osteosarcoma [5]. Evidence regarding the role of RASD1 in hematological cancers is limited and contradictory. Some studies suggest that RASD1 suppresses B-cell proliferation [6], whereas others indicate that it promotes growth in B-cell acute lymphoblastic leukemia [7].

Epigenetic silencing via promoter hypermethylation represents a key mechanism for inactivating tumor suppressor genes. In MM, Nojima et al. reported that RASD1 hypermethylation was associated with dexamethasone resistance [8]. However, the clinical relevance of RASD1 expression and methylation in a well-defined cohort of patients with MM remains underexplored.

This study aimed to comprehensively evaluate RASD1 in MM. The mRNA and protein expression were analyzed in patient samples and correlated with clinical parameters, while the role of DNA methylation in regulating RASD1 expression was investigated. Furthermore, the functional consequences of demethylation on RASD1 expression and cell survival were examined in vitro.

## Materials and methods

### Patient samples and cell culture

Bone marrow aspirates and biopsy tissues were obtained from 26 newly diagnosed patients with MM and 8 healthy controls at the Affiliated Hospital of Zunyi Medical University between June 2019 and October 2020, after obtaining written informed consent. The study was approved by the Ethics Committee of the Affiliated Hospital of Zunyi Medical University (KLLY-2019-209), and was conducted in accordance with the Declaration of Helsinki. All patients with MM were diagnosed according to the International Myeloma Working Group criteria [9]. Peripheral blood mononuclear cells (PBMCs) were isolated using Ficoll density gradient centrifugation, and CD138^+^ plasma cells were subsequently purified from patient PBMCs using magnetic-activated cell sorting. The human MM cell line U266 was obtained from the American Type Culture Collection, and was maintained in Dulbecco’s Modified Eagle Medium supplemented with 10% fetal bovine serum.

### RNA extraction and quantitative real-time PCR (RT-qPCR)

Total RNA was extracted from PBMCs, CD138^+^ cells, and U266 cells using TRIzol reagent, following the manufacturer's protocol. Complementary DNA (cDNA) was synthesized using a reverse transcription kit. RT-qPCR was performed with SYBR Green I Master Mix on a Cobas z480 system. GAPDH served as the endogenous control. The primer sequences for RASD1 were: Forward, 5′-CCA CCG CAA GTT CTA CTC CAT-3′; Reverse, 5′-CCA GGA TGA AAA CGT CTC CTG T-3′. Relative gene expression was calculated using the 2^(-ΔΔCt) method [10].

### DNA extraction and methylation-specific PCR (MSP)

Genomic DNA was extracted using a commercial kit, and bisulfite conversion was performed with the EZ DNA Methylation-Gold™ Kit. MSP was conducted using primers specific for methylated (M) and unmethylated (U) alleles of the RASD1 promoter, as previously described, with modifications [8].

- Methylated Forward: 5′-TTTCGTAGTAGCGTGGATC-3′
- Methylated Reverse: 5′-GTAATCCGAACTCGAACTTT-3′
- Unmethylated Forward: 5′-AGTTTTTGTAGTAGTGTGGATT-3′
- Unmethylated Reverse: 5′-CTCATAATCCAAATCAAACTTT-3′

### Immunohistochemistry (IHC)

Formalin-fixed, paraffin-embedded bone marrow biopsy sections were stained with an anti-RASD1 antibody, following previously described protocols [11]. Staining was evaluated using an immunoreactivity score (IRS), calculated by multiplying the score for the percentage of positive cells (0 = 0%, 1 = 1–25%, 2 = 26–50%, 3 = 51–75%, 4 = 76–100%) by the staining intensity score (0 = negative, 1 = weak, 2 = moderate, 3 = strong). An IRS > 4 was defined as high expression.

Scoring was performed independently by two board-certified pathologists who were blinded to the sample groups (patients with MM vs. healthy controls). Discrepancies, observed in ≤ 10% of cases, were resolved by consensus.

### Cell treatment and apoptosis assay

U266 cells were treated with 50 μM decitabine (DAC) for 48 h. Apoptosis was measured by flow cytometry using Annexin V-YF647A/PI staining, according to the manufacturer's instructions [12].

### Statistical analysis

Data were analyzed using SPSS version 18.0. Group comparisons were performed using Student's t-test or the chi-square test. Receiver operating characteristic curve analysis was performed to assess the diagnostic value of RASD1 mRNA expression. A *P* value < 0.05 was considered statistically significant.

## Results

### RASD1 expression is downregulated in MM

**Fig. 1:**
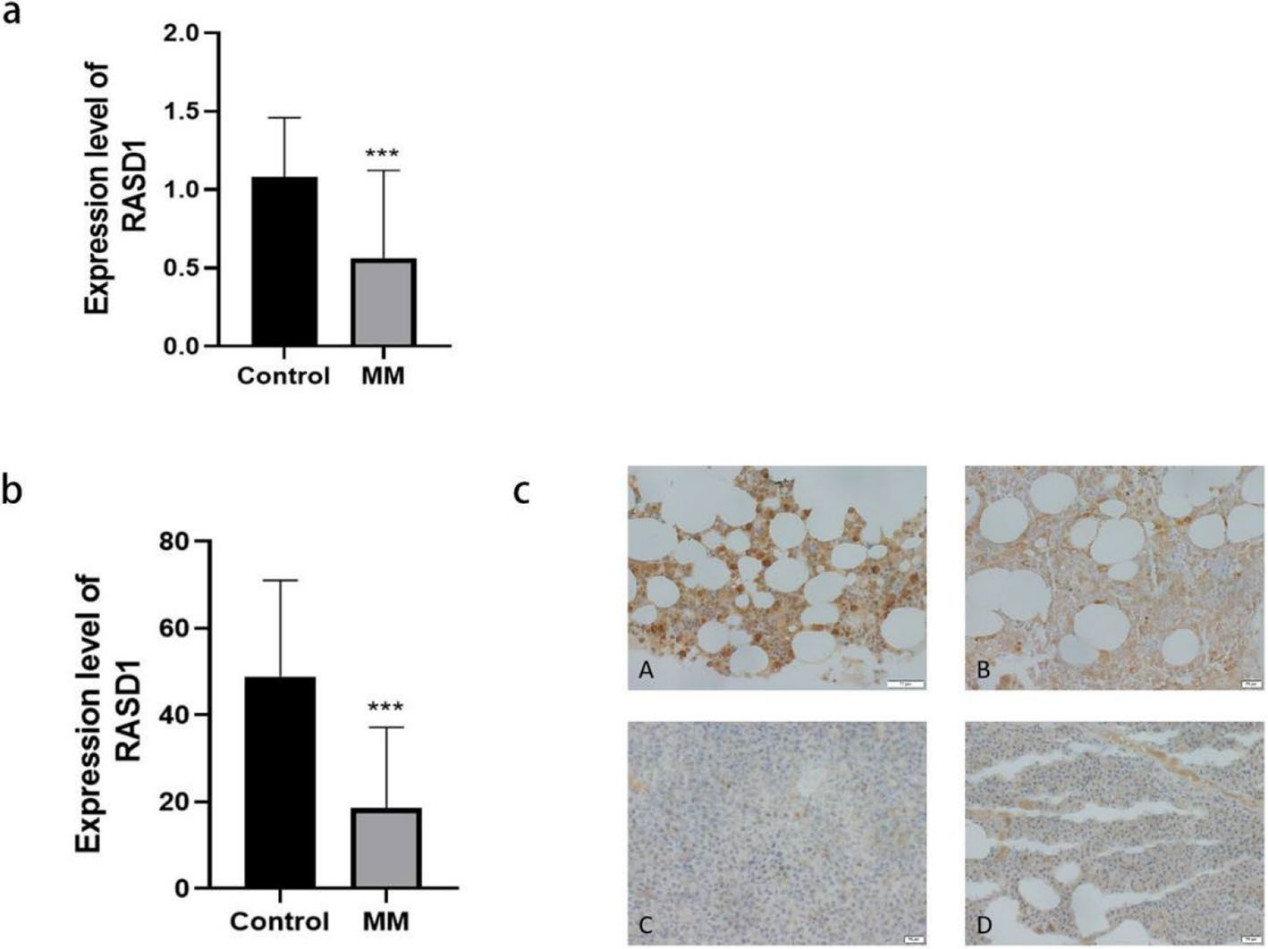
**a** Relative RASD1 mRNA levels (mean ± SD) in patients with MM versus healthy controls (HC), normalized to GAPDH (***P < 0.001). **b** Quantification of RASD1 protein expression (IRS scores, mean ± SD) in patients with MM versus HC (***P < 0.001). **c** Representative IHC images (400× magnification); Panels **A** and **B**: high RASD1 expression in HC bone marrow biopsies; Panels **C** and **D**: low RASD1 expression in bone marrow biopsies from patients with MM

RASD1 mRNA expression was significantly reduced in bone marrow samples from patients with MM (0.42 ± 0.31) compared to healthy controls (1.28 ± 0.67, *P* < 0.001, Fig. 1a). Immunohistochemistry was employed to assess RASD1 protein expression. The results revealed high RASD1 expression in 7 out of 8 (87.5%) healthy controls, with only one case showing low expression. In contrast, high expression was observed in only 6 out of 26 (23.1%) patients with MM, while the remaining 20 cases exhibited low expression. Statistical analysis demonstrated that RASD1 protein expression was significantly reduced in patients with MM compared to healthy controls (*P* < 0.001, Fig. 1b, c).

### Clinical correlations of RASD1 expression

Reduced RASD1 mRNA expression was significantly associated with advanced DS stage (*P* = 0.01) [13], anemia (*P* = 0.04), hypercalcemia (*P* = 0.03), and elevated M-protein levels (*P* = 0.04, Table 1). Similarly, low RASD1 protein expression was correlated with hypercalcemia (*P* = 0.04) and hyperglobulinemia (*P* = 0.04).

**Table 1:** Correlation between RASD1 mRNA Expression and Clinical Characteristics in Patients with MM

Clinical Characteristic	Total (n)	RASD1 Expression		χ ^2^	*P*
		High	Low		
**Gender**					
Male	16	6	10	0.016	0.9
Female	10	4	6		
**Age (years)**					
< 60	18	5	13	2.821	0.09
≥ 60	8	5	3		
**DS Stage**					
Stage I & II	10	3	7	5.987	0.01
Stage III	16	7	9		
**ISS Stage**					
Stage I & II	10	2	8	2.34	1.13
Stage III	16	8	8		
**Anemia**					
Yes	20	9	11	3.869	0.04
No	6	1	5		
**White Blood Cell**					
Normal	24	9	15	0.122	0.72
Abnormal	2	1	1		
**Platelet**					
Normal	15	6	9	0.035	0.85
Abnormal	11	4	7		
**Serum Corrected Calcium (mmol/L)**					
≤ 2.75	19	14	5	4.398	0.03
> 2.75	7	2	5		
**Serum Phosphorus**					
Normal	13	6	7	0.65	0.42
Abnormal	13	4	9		
**M-protein (g/L)**					
< 30	9	2	7	3.313	0.04
≥ 30	17	7	10		

### RASD1 is silenced by promoter hypermethylation

**Fig. 2:**
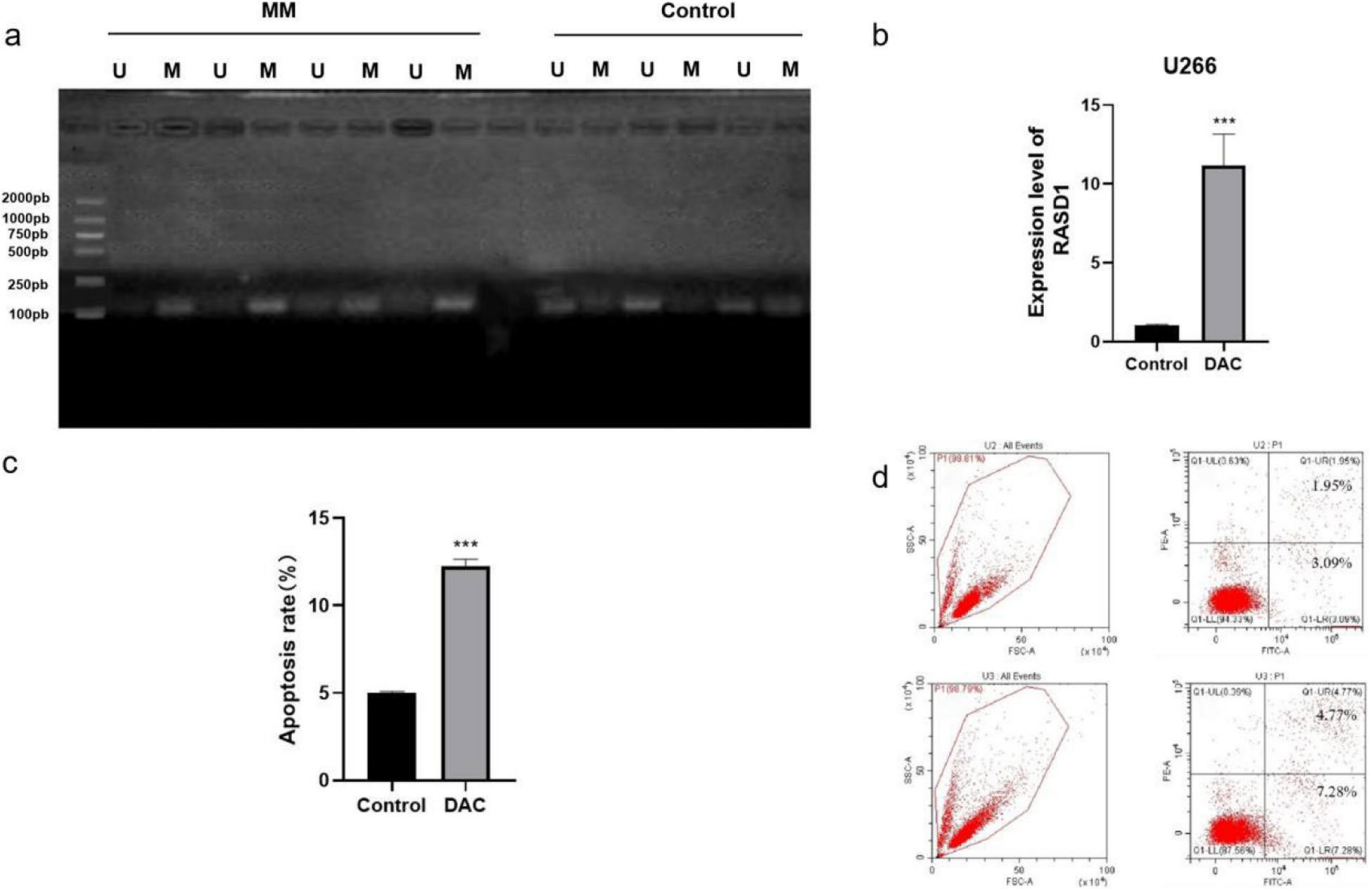
**a** MSP gel electrophoresis showing methylation (M) and unmethylation (U) status in representative samples. **b** RASD1 mRNA expression in U266 cells following DAC treatment. **c** Quantification of apoptosis rates (mean ± SD) in U266 cells (**P < 0.01). **d** Representative flow cytometry plots of apoptosis (Annexin V-YF647A/PI staining); Total apoptosis: 5.04% in the control group and 12.08% in the DAC-treated group

MSP analysis revealed a high frequency of RASD1 promoter methylation in patients with MM, whereas samples from healthy controls were predominantly unmethylated (Fig. 2a). To functionally validate this mechanism, U266 cells were treated with DAC. This treatment significantly restored RASD1 mRNA expression (11.18 ± 1.98 vs. 1.04 ± 0.06; *P* < 0.001; Fig. 2b) and markedly increased apoptosis (12.08% vs. 5.04%, *P* < 0.01; Fig. 2c, d) compared to controls.

## Discussion

Multiple myeloma (MM) remains an incurable plasma cell malignancy despite advances in novel therapeutic agents [1, 2]. The emergence of drug resistance and clonal evolution continues to pose major clinical challenges, highlighting the need for new molecular targets and biomarkers [13]. The present study provides compelling evidence that RASD1 acts as a tumor suppressor gene epigenetically silenced through promoter hypermethylation in MM.

Epigenetic alterations, particularly DNA methylation, have emerged as critical drivers of MM pathogenesis and treatment resistance [14]. Recent studies have demonstrated that aberrant DNA methylation patterns not only silence tumor suppressor genes but also reshape the bone marrow microenvironment [15]. In this context, investigation of RASD1 is particularly relevant because it is located on chromosome 17p11.2, a region frequently altered in hematological malignancies [16].

Significant downregulation of RASD1 expression was observed at both transcriptional and translational levels in patients with MM compared to healthy controls. This finding aligns with recent reports identifying RASD1 as a potential tumor suppressor in other malignancies. For instance, a 2024 study by Ren M. et al. demonstrated that RASD1 restoration inhibits tumor growth in solid tumors through modulation of the MAPK pathway [17].

Mechanistically, DNA hypermethylation was identified as a direct regulator of RASD1 silencing in MM. Treatment with DAC reversed RASD1 expression and induced apoptosis, while MSP analysis confirmed promoter hypermethylation in primary patient samples. These findings are consistent with the current understanding of epigenetic regulation in MM, wherein DNA methyltransferase inhibitors have shown promise in reversing aberrant methylation patterns [18]. Notably, MSP is a qualitative assay; therefore, future studies using quantitative methylation techniques would further delineate the methylation burden of the RASD1 promoter in MM.

Clinically, low RASD1 expression was significantly correlated with established poor prognostic indicators, including advanced DS stage, anemia, and elevated M-protein levels [19, 20]. This clinical relevance is particularly important in the context of recent efforts to develop molecular biomarkers for risk stratification in MM [21]. The association with aggressive disease features suggests that RASD1 methylation status may serve as a valuable biomarker for identifying high-risk patients.

This study has several limitations. First, the sample size was small (26 patients with MM and 8 healthy controls), which limits the generalizability of the conclusions. Second, functional experiments relied on a single MM cell line (U266), and the DAC-induced increase in apoptosis was modest. Third, MSP is a qualitative assay that does not quantify methylation burden. Finally, external validation using publicly available datasets was not conducted. These limitations highlight the need for future studies incorporating larger sample sizes, additional cell lines, quantitative methylation assays, and independent validation to confirm the present findings.

Despite these limitations, the findings provide a foundation for future research. Recent advances in single-cell epigenomics and multiomics approaches present important opportunities to further elucidate the role of RASD1 in MM pathogenesis [22]. Additionally, combining epigenetic therapies with existing MM treatments represents a promising therapeutic avenue that merits further investigation [23].

## Data Availability

Raw data including RT-qPCR Ct values, MSP gel images, and apoptosis flow cytometry plots are available from the corresponding author upon reasonable request.

## References

[1] Kumar SK, Rajkumar SV. The multiple myelomas - current concepts in cytogenetic classification and therapy. Nat Rev Clin Oncol. 2018;15(7):409–21. 29686421 10.1038/s41571-018-0018-y

[2] Rajkumar SV. Multiple myeloma: 2020 update on diagnosis, risk-stratification and management. Am J Hematol. 2020;95(5):548–67. 32212178 10.1002/ajh.25791

[3] Vaidyanathan G, Cismowski MJ, Wang G, et al. The Ras-related protein AGS1/RASD1 suppresses cell growth. Oncogene. 2004;23(34):5858–63. 15184869 10.1038/sj.onc.1207774

[4] Tian J, Duan YX, Bei CY, et al. Calycosin induces apoptosis by upregulation of RASD1 in human breast cancer cells MCF-7. Horm Metab Res. 2013;45(8):593–8. 23609007 10.1055/s-0033-1341510

[5] Both J, Wu T, Ten Asbroek ALMA, et al. Oncogenic properties of candidate oncogenes in chromosome region 17p11.2p12 in human osteosarcoma. Cytogenet Genome Res. 2016;150(1):52–9. 27846620 10.1159/000451046

[6] Lindsey JW. Dexamethasone-induced Ras-related protein 1 is a potential regulatory protein in B lymphocytes. Int Immunol. 2007;19(5):583–90. 17369188 10.1093/intimm/dxm023

[7] Wang S, Wang C, Wang W, et al. High RASD1 transcript levels at diagnosis predicted poor survival in adult B-cell acute lymphoblastic leukemia patients. Leuk Res. 2019;80:26–32. 30925311 10.1016/j.leukres.2019.03.005

[8] Nojima M, Maruyama R, Yasui H. Genomic screening for genes silenced by DNA methylation revealed an association between RASD1 inactivation and dexamethasone resistance in multiple myeloma. Clin Cancer Res. 2009;15(13):4356–64. 19549772 10.1158/1078-0432.CCR-08-3336

[9] Rajkumar SV, Dimopoulos MA, Palumbo A, et al. International myeloma working group updated criteria for the diagnosis of multiple myeloma. Lancet Oncol. 2014;15(12):e538–48. 25439696 10.1016/S1470-2045(14)70442-5

[10] Livak KJ, Schmittgen TD. Analysis of relative gene expression data using real-time quantitative PCR and the 2^(-ΔΔCT) method. Methods. 2001;25(4):402–8. 11846609 10.1006/meth.2001.1262

[11] Shi SR, Shi Y, Taylor CR. Antigen retrieval immunohistochemistry: review and future prospects in research and diagnosis over two decades. J Histochem Cytochem. 2011;59(1):13–32. 21339172 10.1369/jhc.2010.957191 PMC3201121

[12] Rieger AM, Nelson KL, Konowalchuk JD, et al. Modified annexin V/propidium iodide apoptosis assay for accurate assessment of cell death. J Vis Exp. 2011;50:2597. 10.3791/2597 PMC3169266 21540825

[13] Dimopoulos MA, Moreau P, Terpos E, et al. Multiple myeloma: EHA-ESMO Clinical Practice Guidelines for diagnosis, treatment and follow-up. Ann Oncol. 2021;32(3):309–22. 33549387 10.1016/j.annonc.2020.11.014

[14] Agirre X, Castellano G, Pascual M, et al. Whole-epigenome analysis in multiple myeloma reveals DNA hypermethylation of B cell-specific enhancers. Genome Res. 2015;25(4):478-87. 10.1101/gr.180240.114 PMC4381520 25644835

[15] Garcia-Gomez A, Li T, de la Calle-Fabregat C, et al. Targeting aberrant DNA methylation in mesenchymal stromal cells as a treatment for myeloma bone disease. Nat Commun. 2021;12(1):421. 10.1038/s41467-020-20715-x PMC7813865 33462210

[16] Nojima M, Maruyama R, Yasui H, et al. Genomic screening for genes silenced by DNA methylation revealed an association between RASD1 inactivation and dexamethasone resistance in multiple myeloma. Clin Cancer Res. 2009;15(13):4356-64. 10.1158/1078-0432.CCR-08-3336 19549772

[17] Ren M, Pan H, Zhou X, et al. KIAA1429 promotes gastric cancer progression by destabilizing RASD1 mRNA in an m6A-YTHDF2-dependent manner. J Transl Med. 2024;22(1):584. 10.1186/s12967-024-05375-5 PMC11191263 38902717

[18] Heuck CJ. DNA methyltransferase inhibition reverses epigenetically embedded phenotypes in multiple myeloma. Clin Cancer Res. 2021;27(14):3896–907. 24334763 10.1158/1078-0432.CCR-13-1483

[19] Durie BG, Salmon SE. A clinical staging system for multiple myeloma. Correlation of measured myeloma cell mass with presenting clinical features, response to treatment, and survival. Cancer. 1975;36(3):842–54. 1182674 10.1002/1097-0142(197509)36:3<842::aid-cncr2820360303>3.0.co;2-u

[20] Greipp PR, San Miguel J, Durie BG, et al. International staging system for multiple myeloma. J Clin Oncol. 2005;23(15):3412–20. 15809451 10.1200/JCO.2005.04.242

[21] D’Agostino M. A validated gene expression model of high-risk multiple myeloma is defined by deregulated expression of genes mapping to chromosome 1. J Clin Oncol. 2022;40(8):832–45. 10.1182/blood-2006-07-038430 17105813

[22] Tirier SM, Mallm JP, Steiger S, et al. Subclone-specific microenvironmental impact and drug response in refractory multiple myeloma revealed by single-cell transcriptomics. Nat Commun. 2021;12(1):6960. 10.1038/s41467-021-26951-z PMC8630108 34845188

[23] Kumar S, Kats LM, Gruber E. Epigenetic reprogramming in multiple myeloma-Challenges and opportunities. Int J Cancer. 2026;158(2):423-432. 10.1002/ijc.35426 PMC12628036 40171810

